# Evidenzbasierung: Theoriebildung und praktische Umsetzung in Prävention und Gesundheitsförderung

**DOI:** 10.1007/s00103-021-03325-w

**Published:** 2021-04-30

**Authors:** Freia De Bock, Anke Spura

**Affiliations:** 1 Abteilung 2 Effektivität und Effizienz der gesundheitlichen Aufklärung, Bundeszentrale für gesundheitliche Aufklärung (BZgA), Maarweg 149–161, 50825 Köln, Deutschland; 2 Referat 2-24 Fortbildung/Qualifizierung/Hochschulkooperation, Bundeszentrale für gesundheitliche Aufklärung (BZgA), Maarweg 149–161, 50825 Köln, Deutschland

Unsere Gesellschaft steht vor vielfältigen gesundheitlichen Herausforderungen, die mit dem demografischen und gesellschaftlichen Wandel und der Verschiebung der Krankheitslast hin zu nichtübertragbaren chronisch degenerativen und psychischen Erkrankungen verbunden sind. Im Umgang mit diesen Herausforderungen kommt Prävention und Gesundheitsförderung eine besondere Rolle zu. Aber auch im Hinblick auf übertragbare Erkrankungen zeigt jüngst die COVID-19-Pandemie, dass – bei allen Unsicherheiten – evidenzbasiertes, auf einer systematischen Sichtung aller vorhandenen wissenschaftlichen Erkenntnisse verschiedener Disziplinen beruhendes Handeln letztlich der einzige Weg ist, die Herausforderungen zu bewältigen und Vertrauen und Akzeptanz zu sichern.

Im Rahmen des im Jahr 2015 verabschiedeten Präventionsgesetzes (PrävG) hat sich in Deutschland eine neue Dynamik zu Qualität und Evidenz im Feld der Prävention und Gesundheitsförderung entwickelt. Wenn sich das „System Prävention und Gesundheitsförderung“ zunehmend als eine starke Säule des Gesundheitssystems neben Kuration, Rehabilitation und Pflege etablieren soll, müssen Maßnahmen der Prävention und Gesundheitsförderung wirksam sein und darüber hinaus weiteren Kriterien eines evidenzbasierten Vorgehens gerecht werden. Neben rechtlichen Rahmenbedingungen spielen hier das internationale Verständnis von Evidenzbasierung sowie systemisch-ethische Prinzipien eine Rolle.

Diese Dynamik des PrävG und der Pandemie treffen auf ein Feld der Prävention und Gesundheitsförderung in Deutschland, das sehr differenziert und teils fragmentiert organisiert ist. Die Arbeitsgrundlagen, Disziplinen, Professionen und Institutionen sind divers und auch auf der kommunalen Ebene ist ein integriertes Vorgehen oftmals nicht leicht zu erreichen. Es bleibt eine Herausforderung, aussagekräftige Evaluationen zur Wirksamkeit von Maßnahmen durchzuführen und vorliegende wissenschaftliche Erkenntnisse systematisch in der Praxis zu nutzen. Für die Akteurinnen und Akteure aus Praxis und Politik wäre es hilfreich, wenn evidenzbasierte Informationen und Empfehlungen zur Identifikation und Umsetzung geeigneter Maßnahmen zur Verfügung stünden. Diese Empfehlungen müssten in Krisenzeiten dynamisch sein und skaliert werden können.

Allerdings sind rein wissenschaftsbasierte Empfehlungen oft nicht in der Lage, die Praxis tatsächlich zu verändern. Sie müssen übersetzt werden, zum einen in praxistaugliche Konzepte für Entscheidungstragende sowie Akteurinnen und Akteure vor Ort, zum anderen in wirksame Gesundheitsinformationen für die Bevölkerung. Helfen können dabei u. a. Formate wie Datenbanken und vereinfachte Evidenzsynthesen, die in partizipativen Prozessen unter Einbezug der Zielgruppen entwickelt und verfeinert werden. Eine einheitliche Terminologie und Konzeption von Evidenz und evidenzbasiertem Handeln sind hierbei essenziell. Gleichzeitig muss ein zyklischer Lernprozess aus der Anwendung und Umsetzung von Evidenz in der Praxis installiert sein, der praxisrelevante Fragestellungen in die wissenschaftliche Arbeit einspeist.

Das vorliegende Schwerpunktheft will das Verständnis davon, was Evidenzbasierung in der Prävention und Gesundheitsförderung bedeutet, fördern. Es enthält theoretisch-konzeptionelle Operationalisierungen von Evidenzbasierung, Beiträge zur Erhöhung der Praxistauglichkeit und Machbarkeit der Kriterien. Es stellt konkrete Instrumente und Methoden vor, die den Prozess der Evidenzbasierung in der Prävention und Gesundheitsförderung unterstützen können.

Das Schwerpunktheft möchte mit seinen ersten 3 Artikeln Evidenzbasierung in Bezug auf Prävention und Gesundheitsförderung theoretisch-konzeptuell ordnen. Prävention und Gesundheitsförderung sind Kernelemente von Public Health, sodass die theoretisch-konzeptuelle Aufarbeitung auf das breitere Feld der Public Health bezogen ist. *Rehfuess et al.* definieren „evidenzbasierte Public Health“ als „das Fällen von public-health-relevanten Entscheidungen durch die bewusste Integration der jeweils besten verfügbaren wissenschaftlichen Erkenntnisse, der Expertise relevanter Fachleute und Stakeholder und der Werte und Präferenzen der betroffenen Bevölkerung“. Zur praktisch konkreten Bewertung von Public-Health-Maßnahmen schlagen sie einen umfassenden Kriterienkatalog vor.

Der Artikel von *De Bock et al.* greift den Ansatz des ersten Beitrages auf und bringt auf der Grundlage des Memorandums „Evidenzbasierte Prävention und Gesundheitsförderung“ der Bundeszentrale für gesundheitliche Aufklärung (BZgA) einen weiteren konzeptuellen Beitrag in den Diskurs ein. Dieser soll einen Orientierungsrahmen für Akteurinnen und Akteure sowie für Entscheidungstragende vor Ort bzw. auf Landes- und kommunaler Ebene in der Umsetzung, Evaluation und Bewertung evidenzbasierter Maßnahmen bieten. Er fordert aber auch einen Kulturwandel hin zu mehr Evidenzbasierung im Bereich Public Health/öffentliche Gesundheit – äquivalent zur Qualitätsentwicklung in der medizinischen Versorgung.

Evidenzbasierung ist nur dann ein nachhaltig nützliches Qualitätskriterium, wenn Public-Health-Maßnahmen, deren Wirksamkeit mit wissenschaftlichen Mitteln nachgewiesen wurde, auch übertragbar sind. Der Nutzen kann demgemäß erweitert werden, wenn Projekte nicht singulär bleiben, sondern in anderen Kontexten angepasst angewendet werden. In diesem Sinne setzt sich der englischsprachige Beitrag von *Schloemer et al.* mit dem Konzept des Transfers bzw. der Übertragbarkeit von Maßnahmen auseinander und stellt das Modell PIET‑T „Population (P) –Intervention (I) – Environment (E) – Transfer (T) Model of Transferability (PIET-T)“ vor.

Der zweite Teil des Heftes widmet sich methodischen Fragen zur Strukturbildung und Systementwicklung. Akteure, die im kommunalen Setting Programme entwickeln und umsetzen wollen, stehen nicht selten vor einem Problem, wenn sie die Wirksamkeit ihrer Maßnahmen projizieren und evaluieren oder bereits entwickelte Projekte in einen neuen Kontext überführen möchten. *Roßmann et al.* unterbreiten am Beispiel der Interventionsdatenbank des Projektes „Älter werden in Balance“ der BZgA einen methodischen Vorschlag, wie Datenbanken darin unterstützen können, systematisch und kriteriengeleitet Projekterfahrungen zu erfassen und diese anwendungsfreundlich für kommunale Akteure aufbereitet zur Verfügung zu stellen. Kommunen stehen hierbei im Fokus, weil sie einerseits als Akteure von Public Health betrachtet werden können und andererseits zentrale Orte der Umsetzung von Public-Health-Maßnahmen sind.

Ein weiteres Beispiel für Instrumente zur Förderung von Evidenzbasierung stellen *Alayli et al.* mit der Datenbank „Wissen für gesunde Lebenswelten“ des Bündnisses für Gesundheit der gesetzlichen Krankenversicherungen (GKV) vor. Diese basiert auf verschiedenen Arten von Übersichtsarbeiten, die ein probates Mittel zur Generierung von Evidenz sind. Die Datenbank wird fortlaufend erweitert. Die Anwendungsperspektive und damit der Wissenstransfer werden u. a. dadurch ermöglicht, weil Expertinnen und Experten der GKV – auch als adressierte Nutzergruppe – in diesen Prozess einbezogen werden.

Wissenstranslation, die kommunale Akteurinnen und Akteure adressiert und u. a. auch Übersetzung von Wissenschaft für die Praxis umfasst, ist Gegenstand des Beitrages von *Bußkamp et al.* Die Autorinnen stellen die Ergebnisse einer qualitativen Studie vor, die der Frage nachging, welche Bedürfnisse nach evidenzbasierten Informationen bestehen und welche Zugangswege und Barrieren es gibt.

Zu der evidenzbasierten Entwicklung und Umsetzung von Projekten und Maßnahmen für Menschen und Adressatengruppen gehört es aber auch, Menschen und Zielgruppen selbst zu befähigen, in Bezug auf ihre Gesundheitssituation eigenverantwortlich, kompetent und möglichst evidenzbasiert zu handeln. Dabei können sie mit qualitätsgesicherten Gesundheitsinformationen unterstützt werden. *Koch* stellt in seinem Beitrag die in der Guten Praxis Gesundheitsinformation verankerten Grundsätze für evidenzbasierte Gesundheitsinformation vor.

Zur Systementwicklung gehört es auch, dass das Gesundheitswesen als Wissenssystem selbst in Bezug auf Evidenzbasierung kompetent ist. *Trojan et al.* haben Aus‑, Fort- und Weiterbildungsangebote daraufhin gesichtet, ob und inwiefern in ihnen Lehr- und Lerninhalte zum Themenbereich Evidenzbasierung verankert sind. Die Auswertungsergebnisse deuten auf ein erhebliches Steigerungspotenzial hin.

Evidenzbasierung im Praxisfeld Prävention und Gesundheitsförderung kann nur gelingen, wenn entsprechende wissenschaftliche Erkenntnisse vorliegen. *Brandes et al.* stellen die Forschungsverbünde zur Primärprävention und Gesundheitsförderung AEQUIPA, CAPITAL4HEALTH, HLCA, PartKommPlus und SMARTACT vor. Es werden die unterschiedlichen Vorgehensweisen der wissenschaftlichen Evidenzgenerierung beschrieben. In Anbetracht der sehr verschiedenen Forschungsgegenstände, Fragestellungen und methodischen Ansätze zeigt sich u. a. auch die theoretische Herausforderung, diese in eine allgemeine Theorie evidenzbasierter Prävention und Gesundheitsförderung zu fassen. Der Beitrag unterbreitet hierzu einen Vorschlag.

Beim Beitrag von *Osmani und Klug* schauen die Leserinnen und Leser beispielhaft in konkrete Forschungsarbeit hinein, in der es um den präventiven Nutzen von Impfungen gegen humane Papillomaviren (HPV) geht. Es wird deutlich, dass eine gesicherte Evidenzlage und Nutzenbewertung nicht automatisch in praktischer Umsetzung münden, da sie u. a. auf die Akzeptanz in der Bevölkerung angewiesen ist.

In einem System der Prävention und Gesundheitsförderung ist es notwendig, die Zielgruppen oder -regionen mit den höchsten Bedarfen bzw. Risiken zu identifizieren und so die vorhandenen begrenzten Ressourcen, die für Maßnahmen zur Verfügung stehen, effizient einzusetzen. Eine solche Steuerung von Maßnahmen wird am besten datenbasiert aufgesetzt. *Völker et al.* stellen einen Ansatz zur datenbasierten Steuerung von Maßnahmen zur Erhöhung der Impfquote bei den Krankheiten Masern, Mumps und Röteln (MMR) vor. Zentral ist ein geografischer, versorgungsdatenanalytischer Ansatz. Im Ergebnis sind mit einem solchen Verfahren regionale Risikocluster sichtbar, die die Impfversorgung und -kommunikation leiten könnten.

Daten unterstützen Entscheidungsfindung und Steuerung von Maßnahmen auf der Systemebene ebenso wie konkrete Maßnahmen auf der Individualebene. Im Sinne einer Erhöhung der Evidenzbasierung wirbt *Meyer *dafür, den Blick zu weiten für die Chancen, die für Public Health im EU-Raum und in der Zusammenarbeit der Mitgliedstaaten liegen. Jedoch bedarf es hierfür Abstimmungen und die Vereinbarung auf gemeinsame Konzepte und Indikatoren. Der Autor stellt EU-Initiativen vor, die das Ziel verfolgen, Gesundheitsdaten international zu erschließen. Er zeigt damit Wege auf, wie deutsche Akteure Europa als Ressource für evidenzbasierte Public Health nutzen können.

Ihre

Freia De Bock und Anke Spura

